# Implementation of an Immunoassay Based on the MVA-T7pol-Expression System for Rapid Identification of Immunogenic SARS-CoV-2 Antigens: A Proof-of-Concept Study

**DOI:** 10.3390/ijms252010898

**Published:** 2024-10-10

**Authors:** Satendra Kumar, Liangliang Nan, Georgia Kalodimou, Sylvia Jany, Astrid Freudenstein, Christine Brandmüller, Katharina Müller, Philipp Girl, Rosina Ehmann, Wolfgang Guggemos, Michael Seilmaier, Clemens-Martin Wendtner, Asisa Volz, Gerd Sutter, Robert Fux, Alina Tscherne

**Affiliations:** 1 Division of Virology, Department of Veterinary Sciences, Ludwig Maximilians University Munich (LMU Munich), 85764 Oberschleißheim, Germany; satyendra.kumar@lmu.de (S.K.); liangliang.nan@boehringer-ingelheim.com (L.N.); georgia.kalodimou@viro.vetmed.uni-muenchen.de (G.K.);; 2 German Center for Infection Research, Partner Site Munich, 85764 Oberschleißheim, Germanyrosinaehmann@bundeswehr.org (R.E.); 3 Bundeswehr Institute of Microbiology, 80937 Munich, Germany; 4 Chair of Bacteriology and Mycology, Department of Veterinary Sciences, Ludwig Maximilians University Munich (LMU Munich), 85764 Oberschleißheim, Germany; 5 Munich Clinic Schwabing, Academic Teaching Hospital, Ludwig Maximilians University Munich (LMU Munich), 80804 Munich, Germany; wolfgang.guggemos@muenchen-klinik.de (W.G.); michael.seilmaier@muenchen-klinik.de (M.S.); 6 Medical Clinic III, University Hospital, Ludwig Maximilians University Munich (LMU Munich), 80336 Munich, Germany; clemens.wendtner@med.uni-muenchen.de; 7 Institute of Virology, University of Veterinary Medicine Hannover, 30559 Hannover, Germany; asisa.volz@tiho-hannover.de; 8 German Center for Infection Research, Partner Site Hannover-Braunschweig, 30559 Hannover, Germany

**Keywords:** MVA-T7pol, SARS-CoV-2, immunoassay, pandemic preparedness, membrane protein, ORF3a protein, visualization, rapid detection

## Abstract

The emergence of hitherto unknown viral pathogens presents a great challenge for researchers to develop effective therapeutics and vaccines within a short time to avoid an uncontrolled global spread, as seen during the coronavirus disease 2019 (COVID-19) pandemic. Therefore, rapid and simple methods to identify immunogenic antigens as potential therapeutical targets are urgently needed for a better pandemic preparedness. To address this problem, we chose the well-characterized Modified Vaccinia virus Ankara (MVA)-T7pol expression system to establish a workflow to identify immunogens when a new pathogen emerges, generate candidate vaccines, and test their immunogenicity in an animal model. By using this system, we detected severe acute respiratory syndrome (SARS) coronavirus 2 (SARS-CoV-2) nucleoprotein (N)-, and spike (S)-specific antibodies in COVID-19 patient sera, which is in line with the current literature and our observations from previous immunogenicity studies. Furthermore, we detected antibodies directed against the SARS-CoV-2-membrane (M) and -ORF3a proteins in COVID-19 patient sera and aimed to generate recombinant MVA candidate vaccines expressing either the M or ORF3a protein. When testing our candidate vaccines in a prime-boost immunization regimen in humanized *HLA-A2.1-/HLA-DR1-transgenic H-2 class I-/class II*-knockout mice, we were able to demonstrate M- and ORF3a-specific cellular and humoral immune responses. Hence, the established workflow using the MVA-T7pol expression system represents a rapid and efficient tool to identify potential immunogenic antigens and provides a basis for future development of candidate vaccines.

## 1. Introduction

The rapid development and subsequent supply of new therapeutics, effective vaccines, and robust diagnostic assays is essential to stop the global spread not only of circulating but also of newly emerging and hitherto unknown pathogens. However, this development is often limited by the fact that little is known about their immunogenic properties, thus hindering a successful, early response during an outbreak scenario, as seen in the beginning of the COVID-19 pandemic. The uncontrolled and rapid global spread of SARS-CoV-2, with its unprecedented consequences for global trade, economy, and public health, emphasized the need for assays, therapeutics, and vaccines as effective countermeasures. The current licensed vaccines against SARS-CoV-2 are predominantly based on the spike protein, with several studies confirming the immunogenicity and suitability as a target for the development of vaccine and therapeutics (for review, see [1,2,3,4,5]). However, with the emergence of variants of concerns (VOCs), the efficacy of licensed vaccines dramatically decreased [6], highlighting the need for assays to identify additional immunogenic antigens, which may serve as targets for new vaccines and therapeutics. Ideally, the established assays and workflows may not only be used for current outbreaks but may be part of implemented and validated countermeasures for pandemic preparedness. Suitable methods include serological assays, which allow for a rapid identification of potential immunogenic antigens and are (I) time-saving, (II) cheap and cost-efficient, (III) simple and easy to perform, and (IV) handled under BSL-1/2 conditions. To develop a serological assay that meets all these required attributes, we used the well-characterized MVA-T7pol expression system. As a proof-of-concept, we chose SARS-CoV-2 as a reference virus.

SARS-CoV-2, the causative agent of the COVID-19 pandemic, was first detected in December 2019 in Wuhan (China) [7], and the virus spread rapidly all over the world by an efficient human-to-human transmission [8,9]. As of June 2024, over 775 million cases of COVID-19 were reported worldwide, with more than 7 million deaths (WHO). Members of the family *Coronaviridae*, including SARS-CoV-2, encode four structural proteins [10]: envelope (E) protein, nucleocapsid (N) protein, membrane (M) protein, and the spike (S) protein. M, S, and E proteins are located on the virion membrane surface, whereas the N protein is needed for the binding and packing of the viral genome [11]. Additionally, sixteen non-structural proteins (nsp1–16) and nine accessory proteins, namely ORF3a, ORF6, ORF7a, ORF7b, ORF8, ORF9b, ORF9c, and ORF10, have also been identified [11,12,13]. Accessory proteins display different functions, including support during viral infection or transmission in host cells [14], whereas non-structural proteins are involved in viral replication and evasion of the host immune system [15].

MVA, a member of the family *Poxviridae*, was generated by passaging its ancestor, vaccinia virus (VACV) strain Ankara, several hundred times on chicken embryonic fibroblast (CEF) cells, which caused a strict attenuation of the virus. MVA has served for decades as a highly efficient and safe viral vector system through its capability to deliver heterologous viral or bacterial antigens. The highly valuable feature of recombinant MVA to stably insert and express foreign antigens contributed to its further development as a vaccine platform against various infections, including Middle East respiratory syndrome (MERS) [16,17,18,19], SARS [20], and COVID-19 [21,22,23,24,25,26,27].

Besides the stable insertion of foreign genes into the viral genome, another useful feature is the transient and swift expression of target antigens in cell culture systems using the MVA-T7pol expression system [28,29,30]. This system allows for a rapid and highly efficient expression of target antigens without having the laborious process of generating recombinant MVA viruses. Recombinant MVA-T7pol contains a cassette to individually express two different genes, namely lacZ and the bacteriophage T7 RNA polymerase, which are placed under the transcriptional control of either the VACV-specific p11 or p7.5 promoter, respectively, and stably inserted into deletion site II of the MVA genome [29]. Expression of these two genes occur during the replication cycle of MVA-T7pol in the cytoplasm of infected cells.

One major issue when using prokaryotic RNA polymerases in eucaryotic cells is the necessity of the transcribed mRNA to be processed, capped, methylated, and polyadenylated. In general, to obtain unimpaired protein expression driven by the T7 promoter, the T7 RNA polymerase has to be translocated from its synthesis site (cytoplasm) to the nucleus or eukaryotic RNA-modifying enzymes work in the cytoplasm [31]. To overcome this obstacle, the T7 RNA polymerase was introduced into the MVA genome [29], which replicates in the cytoplasm of infected cells and encodes all the enzymes needed for mRNA transcription and processing, including poly(A) polymerase and capping/methylating enzymes. Simultaneous transfection of MVA-T7pol-infected cells (e.g., CEF cells) with a vector plasmid, containing the target antigen under the transcriptional control of the T7 promotor, results in the transient expression of high amounts of protein in the cytoplasm of infected eukaryotic cells. This system has been used successfully in the past to express various proteins such as feline calicivirus capsid protein [32] or pestivirus non-structural proteins [33].

Here, we describe the advantage of using the highly efficient and well-characterized MVA-T7pol expression system for the identification of potential immunogenic antigens of new emerging viruses such as SARS-CoV-2. Furthermore, after successfully identifying immunogenic SARS-CoV-2 antigens using our MVA-T7pol expression system, we generated two recombinant MVA vector viruses, expressing either the M or the ORF3a protein, and tested their immunogenicity in the humanized *HLA-A2.1-/HLA-DR1-transgenic H-2 class I-/class II*-knockout mice using a prime-boost vaccination schedule. Thus, we demonstrated the induction of a substantial amount of M- and ORF3a-specific antibodies and confirmed the published human-specific T-cell epitopes directed against the two antigens, showing the usefulness of the established assay. The obtained results and implemented workflow might be useful in the future to rapidly identify immunogenic antigens of new emerging viruses, which may serve as a basis for the development of effective vaccines and therapeutics.

## 2. Results

### 2.1. In Vitro Characterization of MVA-T7pol

**Figure 1:**
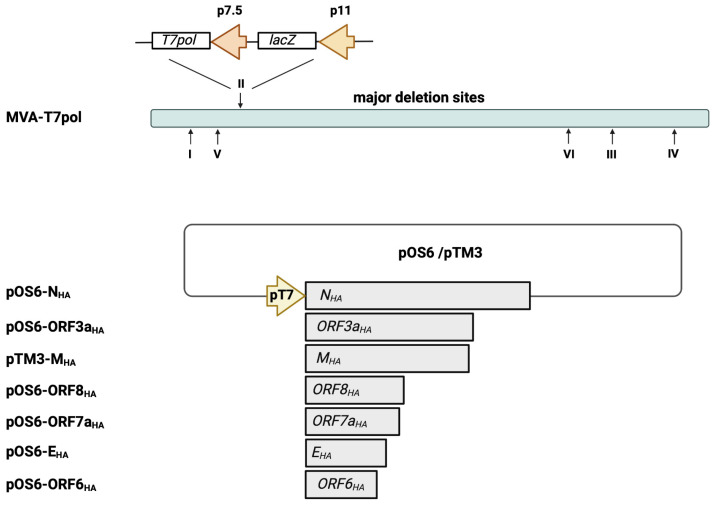
Schematic representation of the MVA-T7pol expression system. The T7 polymerase gene was placed under the control of the vaccinia virus early/late promoter p7.5 and was inserted into MVA deletion site II, as descripted previously [29]. The SARS-CoV-2 gene sequences of N_HA_, E_HA_, M_HA_, ORF3a_HA_, ORF6_HA_, ORF7a_HA_, and ORF8_HA_ were inserted into the vector plasmid pOS6 [29] or pTM3 [34], and expression was placed under transcriptional control of the T7 promoter. The T7-RNA polymerase, which is expressed by recombinant MVA-T7pol during its replication cycle, allows for a transient expression of the SARS-CoV-2 antigens in the cytoplasm of infected cells that are co-transfected with the plasmids pOS6-N_HA_, pOS6-ORF3a_HA_, pTM3-M_HA_, pOS6-ORF8_HA_, pOS6-ORF7a_HA_, pOS6-E_HA_, or pOS-ORF6_HA_. Of note, the target SARS-CoV-2 gene sequences are not inserted into the MVA-T7pol genome. I–VI: major deletion sites of MVA-T7pol. Created with BioRender.com.

The highly efficient MVA-T7pol expression system allows for a transient expression of target antigens that are placed under transcriptional control of the T7 promoter. First, we aimed to characterize in vitro the recombinant MVA-T7pol virus to demonstrate genetic stability and integrity. The expression cassette, containing an RNA T7 polymerase and the lacZ gene, was inserted into deletion site II (Figure 1) [29]. The genetic stability of the recombinant MVA-T7pol virus was confirmed by PCR targeting the six major deletion sites and the *C7L* gene locus of MVA (Supplementary Figure S1a,b). Recombinant MVA-T7pol replicated efficiently to high titers in chicken embryonic fibroblast (CEF) cells but failed to replicate in human HaCat cells (Supplementary Figure S1c). To further characterize the expression pattern of recombinant ß-Galactosidase, total cell lysates from CEF cells infected with MVA-T7pol were analyzed by Western blot. The polyclonal antibody directed against the ß-Galactosidase revealed one prominent protein band that migrated with molecular masses of ~130 kDa (Supplementary Figure S1d). The unimpaired enzymatic activity of ß-Galactosidase was confirmed by staining MVA-T7pol-infected CEF cells (Supplementary Figure S1e). Additionally, we previously generated and characterized an MVA-based candidate vaccine (MVA-S_HA_) against SARS-CoV-2 [22,23,24], which served in the current study as a positive control to express the SARS-CoV-2 S protein.

### 2.2. Transient Expression of Selected SARS-CoV-2 Antigens by MVA-T7Pol

**Figure 2:**
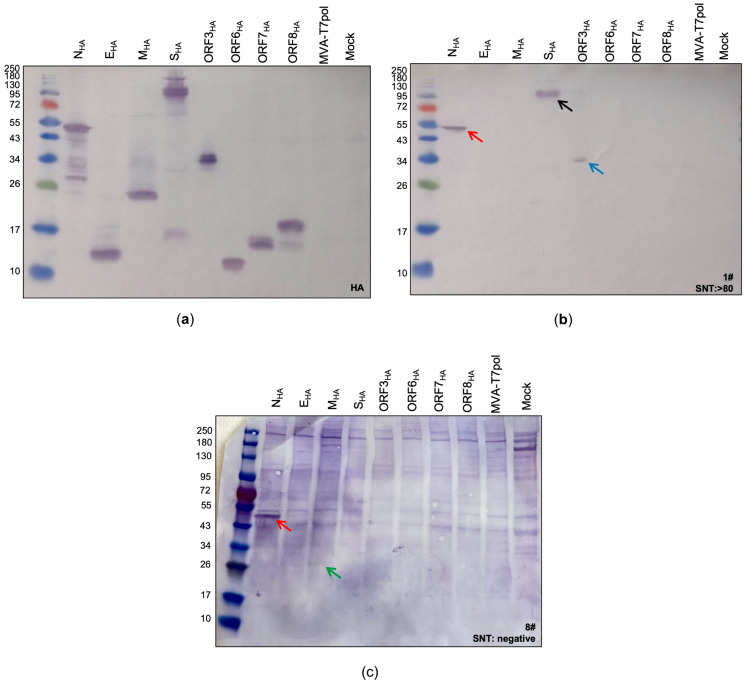
Identification of SARS-CoV-2 proteins expressed by the MVA-T7pol system. To detect the targeted SARS-CoV-2 proteins, CEF cells were infected with recombinant MVA-T7pol at an MOI of 10 and transfected with the vector plasmids pOS6 [29] or pTM3 [34] containing the encoding sequences of the targeted SARS-CoV-2 proteins that were placed under the T7 promoter. To express SARS-CoV-2 spike protein, CEF cells were infected with recombinant MVA-S_HA_ at an MOI of 10. Proteins were separated by SDS-PAGE and analyzed with an antibody directed against the HA-tag (**a**) or by using human serum (**b**,**c**). Non-infected cells (Mock) and cells infected with MVA-T7pol served as controls. Lane 1: N_HA_ (47 kDa); lane 2: E_HA_ (10 kDa); lane 3: M_HA_ (26 kDa); lane 4: S_HA_ (190 kDa + 90 kDa) [24]; lane 5: ORF3a_HA_ (32 kDa); lane 6: ORF6_HA_ (8 kDa); lane 7: ORF7a_HA_ (14 kDa); lane 8: ORF8_HA_ (15 kDa); lane 9: MVA-T7pol; lane 10: non-infected cells (Mock). Red arrow: N protein; black arrow: S protein; blue arrow: ORF3a protein; green arrow: M protein.

Next, we generated transfer plasmids containing the encoding sequences of SARS-CoV-2-E, M, N, ORF3a, ORF6a, ORF7a, and ORF8 proteins placed under the transcriptional control of the T7 promoter (Figure 1). To obtain the desired encoding SARS-CoV-2 sequences, PCR of isolated viral RNA was performed, using oligonucleotide sequences (Supplementary Table S1) designed to amplify the full-length genes and to insert an HA-tag at the C-terminal part and restriction enzyme sites at the C- and N-terminal parts of the SARS-CoV-2 proteins. The oligonucleotide sequences were generated based on published whole-genome sequencing data of SARS-CoV-2, which were already available beginning in 2020 [35]. The amplified SARS-CoV-2 sequences were cloned into the transfer plasmids pTM3 [34] or pOS6 [29], and correct and unimpaired expression of the SARS-CoV-2 target proteins in infected CEF cells was evaluated by Western blotting (Figure 2a), using an antibody targeting the HA-tag.

The standard procedure for immunoblot analysis, including boiling of the lysates at 95 °C for 5–10 min, had to be adapted for the detection of the membrane protein (M_HA_). As described previously [36], boiling lysates containing the membrane protein negatively affects its detection, regardless of whether a reducing agent is used or not (Supplementary Figure S2). Therefore, lysates containing the M_HA_ protein were incubated for 5–10 min at 25 °C before loading on the gel. The bands observed in the subsequent immunoblot perfectly matched to the calculated sizes of N_HA_ (~47 kDa), E_HA_ (~12 kDa), M_HA_ (~26 kDa), ORF3a_HA_ (~34 kDa), ORF6_HA_ (~11 kDa), ORF7a_HA_ (~13 kDa), and ORF8_HA_ (~17 kDa). For S_HA_, two characteristic bands with molecular weights of ~190 kDa and ~90 kDa were observed, representing the full-length S protein and the S2 cleavage product, respectively [24]. After confirming the successful expression of all targeted SARS-CoV-2 antigens, we aimed to screen patient sera for the presence of SARS-CoV-2 specific antibodies as a proof-of-concept.

### 2.3. Screening of COVID-19 Patient Samples for Immunogenic SARS-CoV-2 Antigens

Next, we tested the suitability of the MVA-T7pol system for the detection of immunogenic SARS-CoV-2 antigens in human sera. Therefore, we performed a screening of sera from COVID-19 patients, which had already been tested by ELISA (IgG, IgA) and serum neutralization test (SNT). First, we classified three groups based on the obtained results from the serum neutralization assay: (1) samples #1–3 with SNT >1:80 (group 1), (2) samples #4–6 with SNT = 1:40 (group 2), and (3) samples #7–9 with no detectable neutralizing antibodies (group 3) (Supplementary Table S2). Sera from groups 1 and 2 were also positive for SARS-CoV-2-specific IgA and IgG binding antibodies when tested by ELISA. Sera from group 3 were all negative for SARS-CoV-2-specific IgA and IgG antibodies, except sample #8, which had detectable amounts of IgA antibodies. As expected from the positive ELISA and SNT results, we could identify S- and N-specific antibodies in sera from group 1 and 2 (Supplementary Figure S3). Furthermore, the differences in band sizes observed in our assay indicate that this system might be suitable for semi quantitative analysis, as group 1 sera produced more prominent bands than group 2 sera (Supplementary Figure S3a,b). We could not detect any S-specific antibodies, which tested negative in the neutralization assay. However, two-thirds of the sera from this group showed a weak signal for N (Supplementary Figure S3c). In addition to S- and N- specific antibodies, we identified one patient (#1) showing SARS-CoV-2-ORF3a-specific antibodies (Figure 2b) and another patient (#8) showing SARS-CoV-2-M-specific antibodies (Figure 2c).

In conclusion, we confirmed with our MVA-T7pol expression system that upon infection with SARS-CoV-2, mainly antibodies directly against the spike and nucleoprotein are produced, perfectly matching with the current literature [37,38,39,40,41]. Furthermore, we also identified patient sera showing antibodies against the membrane or ORF3a proteins.

We already generated MVA viruses delivering SARS-CoV-2-S or/and -N proteins, which were successfully evaluated in regard to their immunogenicity and efficacy in several animal models [22,23,24,42,43]. In the current study, we aimed to develop recombinant MVA candidate vaccines expressing the SARS-CoV-2-M and -ORF3a proteins and evaluate their immunogenic properties in humanized *HLA-A2.1-/HLA-DR1-transgenic H-2 class I-/class II*-knockout mice [44].

### 2.4. Generation and Characterization of Recombinant MVA-SARS-CoV-2-M (MVA-M) and MVA-SARS-CoV-2-ORF3a (MVA-ORF3a) Candidate Vaccines

**Figure 3:**
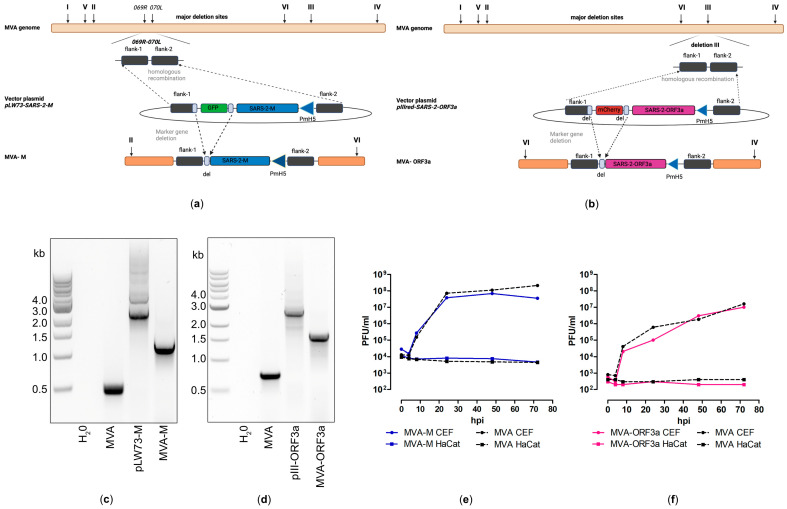
Virological characterization of MVA-SARS-CoV-2-M (MVA-M) and MVA-SARS-CoV-2-ORF3a (MVA-ORF3a). (**a**,**b**) Schematic diagram of the MVA genome with the major deletion sites I to VI. (**a**) The encoding sequence of the full-length SARS-CoV-2 membrane protein (M) was inserted into the vector plasmid pLW73 [45] (pLW73-M). Expression of SARS-CoV-2-M was controlled by the VACV-specific promoter PmH5 [46] and was inserted via homologous recombination between MVA DNA sequences (flank-1, flank-2) adjacent to the intergenomic region between the open reading frames (ORF) of the essential viral genes, *MVA069R* and *MVA070L*, and copies cloned in the MVA vector plasmid pLW73-M. Repetitive sequences served to remove the marker gene GFP by intergenomic homologous recombination (marker gene deletion) to generate MVA-M. (**b**) The deletion III site was targeted to insert the gene sequence encoding SARS-CoV-2-ORF3a under the transcriptional control of VACV promotor PmH5 [46]. Repetitive sequences served to remove the marker gene mCherry by intragenomic homologous recombination (marker gene deletion) to generate MVA-ORF3a. (**c**,**d**) Genetic integrity of MVA-M and MVA-ORF3a. PCR analysis of genomic viral DNA confirmed stable insertion of the SARS-CoV-2-M sequence into the intergenomic region between *069R* and *070L* of the MVA genome and SARS-CoV-2-ORF3a sequence inserted into the deletion III of the MVA genome. (**e**,**f**) Multiple-step growth analysis of recombinant MVA-M, MVA-ORF3a, and non-recombinant MVA (MVA). Recombinant viruses and non-recombinant MVA (MVA) amplified in CEF cells but failed to efficiently grow in human HaCat cells.

**Figure 4:**
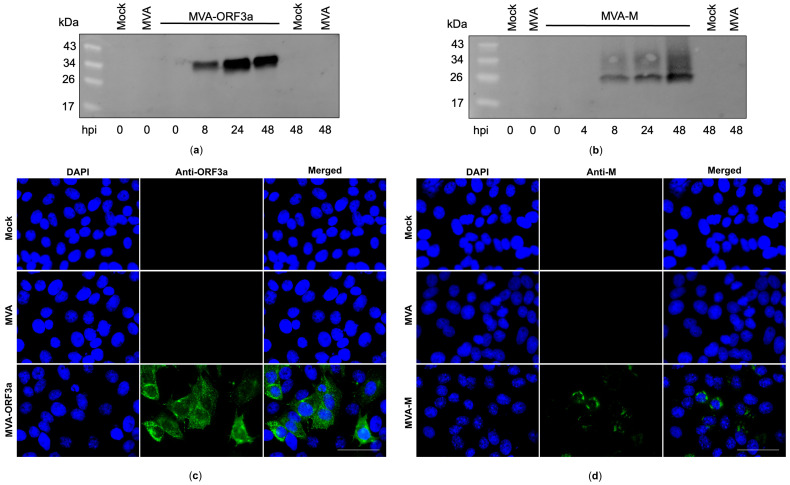
Synthesis of membrane (M) and ORF3a proteins in MVA-M- and ORF3a-infected cells. (**a**,**b**) CEF cells were infected at an MOI of 5, and cell lysates were collected at 0, 4, 8, 24, and 48 h post infection (hpi). Polypeptides in the cell lysates were separated by SDS-PAGE and analyzed with antibodies against the M and ORF3a proteins. (**c**,**d**) Vero cells were infected at an MOI of 0.5 with MVA-M or MVA-ORF3a and fixed with paraformaldehyde after 16 hpi. Permeabilized cells were probed with antibodies against the M and ORF3a proteins. Polyclonal goat anti-mouse secondary antibody was used for M-specific fluorescent staining (green), and polyclonal goat anti-rabbit secondary antibody was used for ORF3a-specific fluorescent staining (green). Cell nuclei were counterstained with DAPI (blue). Scale bar: 50 μm.

Recombinant MVA-M and MVA-ORF3a viruses were generated by introducing the full-length coding sequences of SARS-CoV-2-M and -ORF3a either into the intergenomic region between the two essential genes *MVA-069R* and *MVA-070L* (MVA-M) or deletion site III (MVA-ORF3a) within the MVA genome by homologous recombination (Figure 3a,b). Genetic integrity of the recombinant viruses was confirmed by PCR analysis of viral DNA, demonstrating the site-specific insertion of the SARS-CoV-2-M and SARS-CoV-2-ORF3a gene sequences into the MVA genome and the subsequent removal of the marker genes (mCherry or GFP) during plaque amplification (Figure 3c,d and Figure S4). In addition, we demonstrated the replicative capacity of recombinant MVA-M and MVA-ORF3a in CEF cells and the replicative deficiency in human HaCat cells (Figure 3e,f), confirming the suitability of the recombinant MVA viruses for production at industrial scale and for intendent clinical use, respectively. To determine the expression of recombinant M and ORF3a proteins, CEF cells were infected with recombinant MVA-M, MVA-ORF3a, or non-recombinant MVA (MVA). Cell lysates were prepared at different time points, proteins were separated by SDS-PAGE, and a subsequent immunoblot analysis (Figure 4a,b) was performed. In addition, we stained MVA-M- and MVA-ORF3a-infected Vero cells with M- or ORF3a-specific antibodies and analyzed the expression pattern of the recombinant proteins using fluorescence microscopy (Figure 4c,d).

### 2.5. Recombinant MVA-M and MVA-ORF3a Candidate Vaccines Induced M- and ORF3a-Specific Cellular and Humoral Responses in Humanized HLA-A2.1-/HLA-DR1-Transgenic H-2 Class I-/Class II-Knockout Mice

To further evaluate the immunogenic properties of ORF3a and M, which we observed with the MVA-T7pol expression system, we vaccinated humanized *HLA-A2.1-/HLA-DR1-transgenic H-2 class I-/class II*-knockout mice [44] and screened for cellular and humoral responses. Despite having limitations, the use of transgenic mice that express human HLA is advantageous over wild-type mice as a preclinical model for evaluating the immunogenicity of vaccine candidates based on the human restriction element [44,47,48,49]. Mice were immunized twice intramuscularly with 10^7^ PFU of recombinant MVA-M, MVA-ORF3a, or non-recombinant MVA (MVA), using a three-week interval (Supplementary Figure S5a). Immunizations with both vaccines were well tolerated, and no side effects were observed (Supplementary Figure S5b).

**Figure 5:**
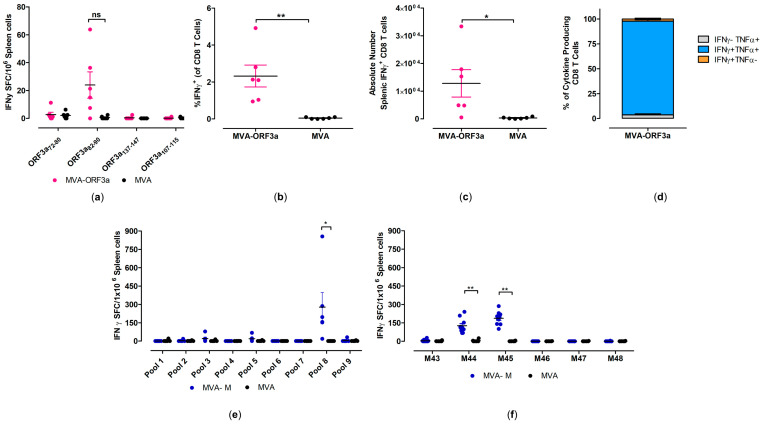
Activation of SARS-CoV-2-M- and SARS-CoV-2-ORF3a-specific CD8 T cells after vaccination with MVA-M and MVA-ORF3a. Groups of *HLA-A2.1-/HLA-DR1-transgenic H-2 class I-/class II-knockout* mice (n = 6–10) were immunized with 10^7^ PFU of MVA-M or MVA-ORF3a via the i.m. route. Mice immunized with non-recombinant MVA (MVA) served as controls. Splenocytes were collected and prepared at day 35 after prime immunization (14 days after booster immunization). Splenocytes were either stimulated with SARS-CoV-2-ORF3a- or SARS-CoV-2-M-specific peptides and were measured by IFN-γ ELISPOT assay (**a**,**e**,**f**) and intracellular cytokines staining (ICS) plus FACS analysis (**b**–**d**). (**a**,**e**,**f**) IFN-γ spot-forming colonies (SFC) measured by ELISPOT assay. (**b**,**c**) IFN-γ producing CD8 T cells measured by FACS analysis. Graphs show the mean frequency and absolute number of IFN-γ+ CD8 T cells. (**d**) Cytokine profile of ORF3a_82-90_ specific CD8 T cells. Graph shows the mean frequency of IFN-γ^−^TNF-α^+^, IFN-γ^+^TNF-α^+^, and IFN-γ^+^TNF-α^−^ cells within the cytokine-positive CD8 T-cell compartment. Bars represent the mean + standard error of the mean (SEM). Differences between group were analyzed by unpaired, two-tailed *t*-test: * *p* < 0.05; ** *p* < 0.01; ns, not significant.

To determine the activation of SARS-CoV-2-M- and SARS-CoV-2-ORF3a-specific cellular immunity following prime-boost vaccination in *HLA-A2.1-/HLA-DR1-transgenic H-2 class I-/class II*-knockout mice, isolated splenocytes were stimulated with ORF3a- and M-specific peptides (Supplementary Tables S3 and S4) and analyzed for cytokine secretion by IFN-γ ELISPOT and ICS-FACS assays (Figure 5).

When testing predicted peptides with isolated splenocytes from mice immunized with 10^7^ PFU of MVA-ORF3a, we detected responses above the background for SARS-CoV-2-ORF3a HLA-A*02:01 epitope ORF3a_82-90_ (NLLLLFVTV) [50,51], with mean numbers of 40 spot-forming cells (SFCs) (Figure 5a). Furthermore, these data aligned well with FACS analysis of T cells stained for intracellular IFN-γ, where we observed higher frequencies (mean of 2.3%) and higher absolute numbers of IFN-γ T cells in immunized animals compared to the control group (Figure 5b,c). We also found that the majority of IFN-γ producing CD8 T cells also co-expressed TNF-α (over 80%) (Figure 5d).

When testing pools of overlapping peptides (Supplementary Table S4) with isolated splenocytes from mice immunized with 10^7^ PFU of MVA-M, we detected responses above the background for one peptide pool (pool 8) (Figure 5e). When we re-stimulated isolated splenocytes with individual peptides from pool 8, we identified two immunodominant SARS-CoV-2-M HLA-A*02:01 epitopes, M_173-187_ (M44, SRTLSYYKLGASQRV) [51,52,53] and M_177-191_ (M45, SYYKLGASQRVAGDS) [51,52,53], with mean numbers of 127 and 188 SFCs, respectively (Figure 5f). The MVA-specific immunodominant CD8 T-cell epitope A6(L)_6-14_ (VLYDEFVTI) [54] served as a control peptide for the detection of MVA-vector-specific CD8 T cells in *HLA-A2.1-/HLA-DR1-transgenic H-2 class I-/class II*-knockout mice (Supplementary Figure S6).

**Figure 6:**
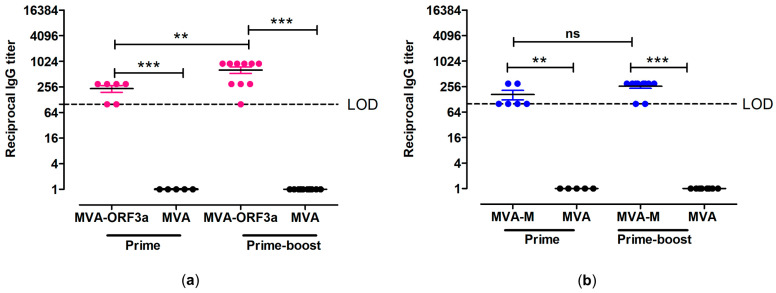
Antigen-specific humoral immunity induced by MVA-ORF3a and MVA-M. (**a**,**b**) Groups of *HLA-A2.1-/HLA-DR1-transgenic H-2 class I-/class II-*knockout mice were immunized with 10^7^ PFU of MVA-M and MVA-ORF3a via the i.m. route. Mice immunized with non-recombinant MVA (MVA) served as controls. Serum samples were collected 18 days after the prime immunization (prime) and 14 days after the booster immunization (prime-boost). Sera were analyzed for (**a**) ORF3a- and (**b**) M-specific IgG by ELISA. Dashed lines represent the limits of detection (LOD). Differences between group were analyzed by unpaired, two-tailed *t*-test: ** *p* < 0.01; *** *p* < 0.001; ns, not significant.

To analyze the humoral immune responses generated by MVA-M and MVA-ORF3a, we screened sera from vaccinated mice for the presence of ORF3a and M antigen-specific IgG binding antibodies. At day 18 after prime immunization, we detected serum IgG antibodies binding to whole recombinant SARS-CoV-2-ORF3a or SARS-CoV-2-M in the sera from 6/6 MVA-ORF3a-vaccinated and 6/6 MVA-M-vaccinated mice by ELISA (Figure 6). Following the booster immunization on day 21, elevated levels of ORF3a- and M-binding IgG antibodies with mean titers of 1:640 for the MVA-ORF3a vaccination group (Figure 6a) and 1:260 for the MVA-M (Figure 6b) vaccination group were observed.

## 3. Discussion

Serological detection assays that are based upon recombinant antigens are commonly applied in diagnostic laboratories. They are especially advantageous for BSL-3 or BSL-4 pathogens because they do not require special containments, unlike whole-virus-based serological assays. Laboratory work involving live viruses always contains the risk of infections and thus, serological assays based on only certain non-contagious parts of the virus are of great importance. Moreover, with the appearance of new emerging viruses, such as MERS-CoV or SARS-CoV-2, an antigen-based serological assay is a rapid and safe method to identify and characterize the immunological properties of these hitherto unknown pathogens.

In this study, we established a workflow for an antigen-based serological assay using the well-characterized MVA-T7pol expression system to identify potential immunogenic antigens of new emerging viruses (Supplementary Figure S7).

We used SARS-CoV-2 as an example for a new emerging virus, with the aim to identify potential immunogenic antigens, followed by the development of novel candidate vaccines based on the obtained results from screening patient sera. The replication-deficient MVA-T7pol virus displays a solid and powerful expression system to study the functionality of recombinant genes. As opposed to replicative-efficient VACV-T7pol expression systems, the absence of viral replication in cells of mammalian origin represents a substantial safety benefit. Moreover, the MVA-T7pol expression system allows for the production of large quantities of foreign proteins, without the need to construct and isolate virus recombinants. Using the MVA-T7pol expression system, we expressed three structural (N, E, and M) and four accessory proteins (ORF8, ORF7, ORF6, and ORF3a), which were further investigated with a systematic Western blot approach using COVID-19 patient sera from 2020. To confirm correct expression of the viral proteins, and because SARS-CoV-2-specific antibodies were not available for all proteins, we added an HA-tag at the C-terminal part of the proteins. In addition, this enabled us to balance the concentration of the proteins of interest to guarantee comparable immunoblot signals.

In our experiments, we screened COVID-19 patient sera for the presence of antibodies against SARS-CoV-2 antigens and mainly found antibodies directed against the spike, nucleocapsid, membrane, and ORF3a protein. Signals associated with the N protein were visualized faster than the other proteins when incubating with the patient sera. Moreover, N-specific but not S-specific antibodies were detected in two sera (#7 and #8). This suggests that N-based serological assays may have a higher sensitivity, as others found previously for SARS-CoV-1 and SARS-CoV-2 [55,56]. It has been shown that N-specific antibodies are detectable earlier than S-specific antibodies for SARS-CoV-2, SARS-CoV-1, and other human coronaviruses [37,57]. Earlier studies pointed out that serological assays based on the nucleoprotein might lead to false-positive results, as SARS-CoV-2 nucleoprotein shares conserved areas with other human coronavirus nucleoproteins [58]. In contrast, van Elslande et al. [59] demonstrated that diagnostic assays screening for N-specific antibodies showed superior specificity. However, the aim of this study was not to evaluate characteristics of the MVA-T7pol expression system as a clinical diagnostic application; in particular, because of the small number of tested sera, any conclusion would not be appropriate.

The results obtained by ELISA (S-specific IgG and IgA) correlated well with both the SNT data and the results from our S-specific immunoblot analysis. We assume therefore, that detecting S-specific antibodies by Western blotting may be an indicator for neutralizing antibody responses, as published by others before [60].

Commercial serological assays target only certain viral antigens, such as, in the case of SARS-CoV-2, mainly S and N proteins. Other viral proteins that might be of interest for the development of vaccines or therapeutics are excluded. By using the MVA-T7pol expression system, we stably expressed other viral proteins, and through this, we identified two patient sera showing antibodies directed against the M and ORF3a proteins. M protein is synthesized in the host cells, present in the endoplasmic reticulum–Golgi compartment, provides a platform for the recruitment of other structural proteins, and plays an important role during virus assembly [61,62,63]. Zeng et al. [63] demonstrated in sera of SARS patients elevated levels of ORF3a-specific antibodies, indicating a target for the development of effective therapeutics or vaccines or therapeutics [64]. Recently published studies on lysosomal exocytosis demonstrated that SARS-CoV-2-ORF3a promotes lysosomal targeting of exocytosis-related SNARE proteins and the BORC-ARL8b complex [65]. In addition, it has been shown that ORF3a induces necrotic cell death and lysosomal damage [66,67]; moreover, Wang et al. [68] reported that ORF3a inhibits the interferon signaling pathway via the upregulation of suppressor targeting cytokine signaling 1 [69,70,71]. In the current study, we detected one COVID-19 patient showing ORF3a-specific antibodies, confirming the observations of Camerini et al. [71], who detected anti-ORF3a antibodies using a microarray based on multiple coronavirus proteins. However, due to the limited number of tested serum samples, these findings need be further evaluated.

Furthermore, we assume that the MVA-T7pol expression system may be suitable for further functional analysis of target proteins. For example, when analyzing the humoral immune response against the SARS-CoV-2 membrane protein, we only saw faint bands when we followed the standard procedures for our immunoblot assays. However, when the boiling step of the sample preparation was omitted, the signals increased significantly, indicating a thermal aggregation property comparable to SARS-CoV-1-M [36].

Within a very short time after the first emergence of SARS-CoV-2, there has been a drastic increase in the number of developed diagnostic assays to confirm COVID-19 infections [72,73,74,75,76]. As a classical method based on antigen-antibody response, Western blot was the basis of serological assays to provide the accuracy of a pre-screening test for Lyme disease and HIV infection [77]. Antibodies from positive samples were visualized in the form of specific bands to the naked eye in our study, making the detection convenient and promising to apply in the future when new pathogenic viruses emerge. The currently available vaccines mainly deliver the S antigen, and simultaneous detection of nucleoprotein- and spike-specific antibodies may help to confirm SARS-CoV-2 natural infection in vaccinated individuals.

Based on our observations with the MVA-T7pol expression system, we generated two recombinant MVA vector viruses expressing either SARS-CoV-2-ORF3a (MVA-ORF3a) or -M (MVA-M) and tested their immunogenicity in humanized *HLA-A2.1-/HLA-DR1-transgenic H-2 class I-/class II*-knockout mice. After prime-boost immunization with MVA-ORF3a and MVA-M, we were able to detect ORF3a- and M-specific cellular and humoral immune responses. The latter fits with our observations when using the MVA-T7pol expression system, and in addition, the identified M- and ORF3a-specific human T-cell epitopes are in line with the current literature [50,51,53], thus confirming the suitability to identify the immunogenic properties of proteins with our described method.

The current study has limitations. We did not test the protective efficacy of our two MVA candidate vaccines in a prime-boost challenge model, as the aim of this study was the establishment of a serological assay to identify immunogenic antigens. In future studies, the question of the efficacy of our two candidate vaccines will be addressed.

## 4. Materials and Methods

**Cell culture**. Primary chicken embryonic fibroblast (CEF) cells were prepared [78] from 11-day-old chicken embryos (SPP eggs, VALO, Cuxhaven, Germany) using recombinant trypsin (Tryple TM, Thermo Fisher Scientific, Planegg, Germany) and were maintained in Dulbecco’s Modified Eagle’s Medium (DMEM) (Sigma-Aldrich, Taufkirchen, Germany) with 10% heat-inactivated fetal bovine serum (FBS) (Thermo Fisher Scientific, Planegg, Germany) and 1% MEM non-essential amino acid solution (Sigma-Aldrich, Taufkirchen, Germany). Vero cells (ATCC CCL-81) (ATCC, Manassas, VA, US) were maintained in DMEM, 10% FBS, and 1% MEM non-essential amino acid solution (Sigma-Aldrich, Taufkirchen, Germany). Human HaCat cells (CLS Cell lines Service, Eppelheim, Germany) were maintained in DMEM, 10% FBS, 1% HEPES solution (Sigma-Aldrich, Taufkirchen, Germany), and 1% MEM non-essential amino acid solution. All cells were cultivated at 37 °C and 5% CO_2_.

**COVID-19 patient samples**. The human sera used in this study to validate the MVA-T7pol expression system were kindly supplied by the Bundeswehr Institute of Microbiology in Munich (80937 Munich, Germany) and Munich Clinic Schwabing (Academic Teaching Hospital, 80804 Munich, Germany). The serum panel comprised sera from individuals who tested positive for COVID-19 in 2020 and samples from individuals suspected for COVID-19 infection in 2020 but confirmed negative. All serum samples were heat-inactivated at 56 °C for 15–30 min.

**Plasmid Construction**. Viral RNA of SARS-CoV-2 (isolate MUC IMB-1, provided by the Bundeswehr Institute of Microbiology in Munich, 80937 Munich, Germany) was purified using the QiaAmp Viral RNA extraction kit (Qiagen, Hilden, Germany) according to the manufacturer’s recommendations. Complementary DNA (cDNA) was obtained by using the SuperScript VILO kit (Life Technologies, Darmstadt, Germany) according to the manufacturer´s instructions. The generated cDNA served to amplify the selected full-length SARS-CoV-2 sequences (ORF8, ORF7, ORF6, ORF3a, M, N, and E) by PCR, and additionally, restriction enzyme recognition sequences and a C-terminal HA-tag were included in all sequences (Supplementary Table S1). PCR amplicons were cloned into the plasmids pTM3 [34], or pOS6 [29] was placed under the transcriptional control of the T7 promoter. Correct insertion of the seven target genes was confirmed by sequencing analysis (Eurofins, Ebersberg, Germany). The coding sequences of full-length SARS-CoV-2-M and -ORF3a proteins from virus isolate Wuhan HU-1 (GenBank accession no. MN908947.1) were modified in silico to remove runs of guanines or cytosine and terminal signals of vaccinia virus (VACV)-specific early transcription. The modified M and ORF3a cDNAs were generated by gene synthesis (Eurofins, Ebersberg, Germany) and were cloned into the MVA transfer plasmid pLW73 [45] or pIIIH5red to obtain the MVA expression plasmids pLW73-SARS-2-M (pLW73-M) and pIIIH5red-SARS-2-ORF3a (pIIIH5red-ORF3a), respectively. Expression of the recombinant proteins was regulated by the synthetic VACV-specific early/late promoter PmH5 [46].

**Generation of recombinant viruses**. The generation of recombinant MVA expressing the T7 RNA polymerase/ß-Galactosidase (MVA-T7pol) and recombinant MVA expressing SARS-CoV-2-S (MVA-S_HA_) was described previously [24,29,78]. MVA vector viruses expressing SARS-CoV-2-M or -ORF3a were obtained using well-established protocols for vaccine development, as described in previous studies [78]. In brief, 90–95% confluent monolayers of CEF cells were grown in six-well tissue culture plates (Sarstedt, Nümbrecht, Germany), infected with non-recombinant MVA (clonal isolate MVA-F6-sfMR) at a multiplicity of infection (MOI) of 0.05, and transfected with MVA transfer plasmids pLW73-M or pIIIH5red-ORF3a using X-tremeGENE HP transfection reagents (Roche diagnostics, Penzberg, Germany) according to the manufacturer´s instructions. Subsequently, cell cultures were collected, and recombinant MVA viruses were clonally isolated by serial rounds of plaque purification on CEF cell monolayer by monitoring for transient co-expression of the green or red fluorescent marker proteins GFP and mCherry. To prepare vaccine stocks, recombinant MVA viruses were amplified on CEF cell monolayers grown in T175 tissue culture flasks (Sarstedt, Nümbrecht, Germany), purified by ultracentrifugation through 36% sucrose (Sigma-Aldrich, Taufkirchen, Germany) cushion, and reconstituted to high-titer vaccine stock preparation in Tris-buffer saline (pH 7.4) (PanReac AppliChem, Darmstadt, Germany). Plaque-forming units (PFU) were counted to determine viral titers [78].

**In vitro characterization of recombinant MVA-T7pol, MVA-SARS-CoV-2-M (MVA-M), and MVA-SARS-CoV-2-ORF3a (MVA-ORF3a) viruses**. The genetic identity and stability of recombinant viruses were confirmed by polymerase chain reaction (PCR) using isolated viral DNA. PCR analysis was performed using oligonucleotide sequences targeting the six major deletions sites and the inserted gene sequences [78]. In addition, replicative capacity of recombinant MVA viruses was tested in multiple-step growth experiments on monolayers of CEF and HaCat cells. Cells were infected at an MOI of 0.05; collected at 0, 4, 8, 24, 48, and 72 h post infection (hpi); and titrated on CEF cell monolayers to determine the infectivity in cell lysates in PFU [78]. Additionally, the unimpaired enzymatic activity of ß-Galactosidase was determined using the ß-Gal Staining Kit (Roche diagnostics, Penzberg, Germany) according to the manufacturer’s instructions.

**Western blot analysis**. To detect the SARS-CoV-2 spike protein (S_HA_), CEF cells were infected with MVA-S_HA_ at an MOI of 10, and cells were collected and lysed with lysis buffer (1% Triton X-100 (Sigma-Aldrich, Taufkirchen, Germany), 1 M NaCl (Carl Roth, Karlsruhe, Germany), 25 mM Tris) at 24 hpi. To determine the unimpaired expression of the SARS-CoV-2 target proteins, CEF cells were infected with recombinant MVA-T7pol at an MOI of 10 and co-transfected with the expression plasmids pTM3 [34] or pOS6 [29] (1 µg) using X-tremeGENE HP DNA Transfection Reagent, following the manufacturer´s instructions (Roche Diagnostics, Penzberg, Germany). Proteins were extracted, quantified using a BCA assay (Thermo Fisher Scientific, Planegg, Germany) according to the manufacturer´s instructions, and mixed with sample buffer (Bio-Rad, Feldkirchen, Germany). Samples were boiled at 95 °C for 5–10 min, with the exception of the M_HA_ lysates [36]. Afterwards, proteins were separated via SDS-PAGE (Bio-Rad, Feldkirchen, Germany) and transferred onto nitrocellulose membranes (GE Healthcare, Freiburg, Germany). Membranes were blocked with blocking buffer (5% milk in PBS/0.05% Tween20 (PBST)) (Sigma-Aldrich, Taufkirchen, Germany) for 1 h at 25 °C and incubated with an antibody targeting the HA-tag (clone mAb 2-2.2.14; 1:8000 diluted in blocking buffer (Thermo Fisher Scientific, Planegg, Germany) for 1–2 h at 25 °C. Subsequently, blots were washed thrice with PBST, followed by incubation with a secondary goat anti-mouse IgG/HRP antibody (1:5000 in blocking buffer, Agilent Dako, Glostrup, Denmark) for 1–2 h at 25 °C. Subsequently, blots were rinsed thrice with PBST, covered with TrueBlue™ Peroxidase Substrate (SeraCare Life Sciences, Gaithersburg, MD, USA), and incubated until color change was observed. The reaction was stopped by washing the membranes once with dH_2_O.

To confirm unimpaired ORF3a and M protein expression over time, CEF cells were infected at an MOI of 5 with recombinant MVA-M, MVA-ORF3a, or non-recombinant MVA, or they remained uninfected (mock). Cell lysates were prepared after 0, 8, 24, and 48 hpi using NP-40 lysis buffer (50 mM Tris-HCl (PanReac AppliChem, Darmstadt, Germany), 150 mM NaCl (Carl Roth, Karlsruhe, Germany), 1% NP-40 (Thermo Fisher Scientific, Planegg, Germany)) to extract the proteins. Protein samples were stored at −80 °C until further use. Proteins were separated by SDS-PAGE (Bio-Rad, Feldkirchen, Germany), followed by transfer onto nitrocellulose membrane by electroblotting. Membranes were blocked in PBS containing 5% milk (Sigma-Aldrich, Taufkirchen, Germany), followed by incubation overnight at 4 °C with primary antibodies targeting SARS-CoV-2-M (1:1000, Genetex, Alton Pkwy Irvine, CA, USA) or SARS-CoV-2-ORF3a (1:1000, Genetex, Alton Pkwy Irvine, CA, USA). Subsequently, membranes were washed with PBST and incubated for 1 h at room temperature with goat anti-mouse IgG/HRP (Agilent Technologies, Glostrup, Denmark) or goat anti-rabbit/HRP (Cell Signaling Technologies, Danvers, MA, USA). Membranes were washed and developed with SuperSignal^®^ West Dura Extended Duration substrate (Thermo Fisher Scientific, Planegg, Germany). A ChemiDoc MP Imaging System (Bio-Rad, Munich, Germany) was used to visualize chemiluminescence.

To confirm unimpaired expression of ß-Galactosidase, CEF cells were infected at an MOI of 5 with recombinant MVA-T7pol or non-recombinant MVA (MVA), or they remained uninfected (mock). Cell lysates were prepared at 20 hpi using NP-40 lysis buffer to extract the proteins. Protein samples were stored at −80 °C until further use. Proteins were separated by SDS-PAGE, followed by transfer onto nitrocellulose membrane by electroblotting. Membranes were blocked in PBS containing 5% milk, followed by incubation overnight at 4 °C with a primary antibody targeting ß-Galactosidase (1:1000, Genetex, Alton Pkwy Irvine, CA, USA). Subsequently, membranes were washed with PBST and incubated for 1 h at room temperature with goat anti-rabbit/HRP. Membranes were washed and developed with SuperSignal^®^ West Dura Extended Duration substrate. A ChemiDoc MP Imaging System was used to visualize chemiluminescence.

**Systematic immunoblot analysis to confirm SARS-CoV-2 antibodies in COVID-19 patient sera**. First, 4× Laemmli sample buffer (Bio-Rad, Munich, Germany) was added to the cell lysates, which were subsequently boiled at 95 °C for 5–10 min. Then, 35 µg protein/well was loaded, and electrophoresis was conducted at 80–120 V for 70 min. Subsequently, separated proteins were transferred onto nitrocellulose membranes for 70 min at 120V. Blots were incubated with blocking buffer (5% milk dissolved in PBST) for 1–3 h at 25 °C and subsequently probed with the COVID-19 patient sera (1:200 dilution) for 16 h at 4 °C. Blots were rinsed three times with PBST and probed with a rabbit anti-human IgG/HRP antibody (1:2000 in blocking buffer, for 1 h at 25 °C. Blots were rinsed three times with PBST and incubated with TrueBlue™ Peroxidase Substrate). The reaction was stopped by washing the membranes with dH_2_O.

**Serum neutralization test (SNT)**. Neutralizing antibody (nAb) titers in human patient sera were determined using an in-house serum neutralization test as described previously [79]. Briefly, serum samples (duplicates) were diluted in 96-well tissue culture plates (Greiner bio-one, Frickenhausen, Germany) using MEM, supplemented with antibiotic-antimycotic solution and MEM non-essential amino acid solution (all Invitrogen, Thermo Fisher Scientific, Darmstadt, Germany). Next, 100 TCID_50_ of strain MUC IMB-1 was incubated with the diluted serum samples for 1 h at 37 °C (5% CO_2_) before Vero E6 cells (1 × 10^4^ cells/50 µL) were transferred to all wells. Cells were incubated for 72 h, and afterwards, supernatants were discarded, and cells were fixed with 3% formalin/PBS (Sigma-Aldrich, Taufkirchen, Germany), and stained with crystal violet (0.1%) (Thermo Fisher Scientific, Planegg, Germany). The nAb titer corresponded to the reciprocal of the highest serum dilution demonstrating complete inhibition of cytopathic effect (CPE). A virus re-titration was conducted on every plate.

**Vaccination experiments in mice**. Specific-pathogen-free 6- to 10-week-old humanized *HLA-A2.1-/HLA-DR1-transgenic H-2 class I-/class II*-knockout mice (in-house bred) [44] were kept in isolated cage units (Techniplast, Hohenpeißenberg, Germany) with free access to water and food. The immunization experiments were approved by the Government of Upper Bavaria, Munich, Germany. Groups of mice were immunized twice over a 21-day interval with recombinant MVA viruses at a dose of 10^7^ PFU in the hind legs (intramuscularly). Mice immunized with non-recombinant MVA (MVA) at a dose of 10^7^ PFU served as control. After immunization, mice were monitored daily for well-being and health constitution using a score sheet. In addition, the weight of mice was checked daily. Blood samples were collected on days 18 and 35. Coagulated blood was centrifuged at 2000× *g* for 10 min to separate serum.

**T-cell analysis by enzyme-linked immunospot assay (ELISPOT)**. Mice were sacrificed at day 35 post prime immunization, and splenocytes were isolated. Spleens were passed through a 70 µm stainer (Falcon^®^, Sigma-Aldrich, Taufkirchen, Germany) and incubated with Red Blood Cell Lysis Buffer (Sigma-Aldrich, Taufkirchen, Germany). Cells were washed and resuspended in RPMI-10 (RPMI 1640 medium containing 10% FBS, 1% Penicillin-Streptomycin, 1% HEPES; Sigma-Aldrich, Taufkirchen, Germany). ELISPOT assay (Mabtech ELISpot kit for mouse IFN-γ, Stockholm, Sweden) was performed following the manufacturer´s instructions to measure IFN-γ producing T cells. Then, 2 × 10^5^ splenocytes/well were seeded in 96-well plates and stimulated with individual peptides or pools containing overlapping peptides (2 µg/mL diluted in RPMI-10) (Supplementary Tables S3 and S4). Non-stimulated cells and cells stimulated with phorbol myristate acetate (PMA)/ionomycin (Sigma-Aldrich, Taufkirchen, Germany) or VACV peptide VLYDEFVTI (A6(L)_6-14_) [54] served as controls. After incubation at 37 °C and 5% CO_2_ for 48 h, plates were stained following the manufacturer´s instructions. Spots were counted and analyzed by using an automated ELISPOT plate reader (Bioreader^®^ 7000 V, BIOSYS Scientific Devices GmbH, Karben, Germany).

**T-cell analysis by intracellular cytokine staining (ICS)**. The detailed protocol for intracellular cytokine staining (ICS) was described previously [80]. In brief, mice were sacrificed at day 35 post prime immunization, and splenocytes were isolated as described above. Afterwards, 10^6^ cells/well were seeded and stimulated with individual peptides (8 µg/mL diluted in RPMI-10) (Supplementary Table S3). Non-stimulated cells and cells stimulated with PMA/ionomycin (Sigma-Aldrich, Taufkirchen, Germany) or VACV peptide VLYDEFVTI (A6(L)_6-14_) [54] served as controls. Then, brefeldin A was added, and cells were incubated for additional 4 h. After stimulation, cells were stained with anti-mouse CD3 phycoerithrin (PE)-Cy7 (clone 17A2, 1:100, Biolegend, San Diego, CA, USA), anti-mouse CD4 Brilliant Violet 421 (clone GK1.5, 1:600, Biolegend, San Diego, CA, USA), anti-mouse CD8α Alexa Fluor 488 (clone 53-6.8, 1:300, Biolegend, San Diego, CA, USA), and purified CD16/CD32 (Fc block; clone 93, 1:500, Biolegend, San Diego, CA, USA) (Supplementary Table S5). Subsequently, cells were washed, fixed, and permeabilized with Perm Wash buffer (Biolegend, San Diego, CA, USA). Finally, cells were intracellularly stained with anti-mouse IFN-γ allophycocyanin (APC) (Biolegend, San Diego, CA, USA) and anti-mouse TNF-α PE (Biolegend, San Diego, CA, USA). Data were acquired by NovoCyte Quanteon flow cytometer (Agilent Technologies, Waldbronn, Germany) and analyzed using FlowJo software, version 10.10 (FlowJo LLC, BD Life Sciences, Ashland, OR, USA).

**SARS-CoV-2 antigen-specific IgA and IgG enzyme-linked immunosorbent assay (ELISA)**. Anti-SARS-CoV-2 IgA and IgG ELISAs were performed at the Bundeswehr Institute of Microbiology using a commercial ELISA, following the manufacturer’s instructions (Euroimmun, Lübeck, Germany). Anti-SARS-CoV-2-M- and -ORF3a-specific serum IgG titers in the serum of immunized mice were analyzed by in-house ELISAs. Flat-bottom 96-well ELISA plates (F96 MaxiSORP Nunc Immuno, Thermo Fisher Scientific, Planneg, Germany) were coated with 50 ng/well of recombinant SARS-CoV-2-ORF3a (Proteogenix, Schiltigheim, France) or 100 ng/well of recombinant SARS-CoV-2-M (Proteogenix, Schiltigheim, France) protein overnight at 4 °C. Mouse sera were serially diluted in PBS/1% BSA (Sigma-Aldrich, Taufkirchen, Germany), transferred to the pre-coated ELISA plates, and incubated for 1 h at 37 °C. Afterwards, plates were washed trice, probed with a secondary goat anti-mouse IgG/HRP, diluted in PBS/1% BSA, incubated for 1 h at 37 °C, and subsequently developed with 3,3′,5,5′-tetramethylbenzidine (TMB) (Sigma-Aldrich, Taufkirchen, Germany) as chromogenic substrate. The reaction was stopped by adding a stop solution (Sigma-Aldrich, Taufkirchen, Germany). Absorbance was measured at 450 nm with a 620 nm reference wavelength, and the obtained data were normalized using a positive control. The cut-off value for positive serum samples was determined by calculating the mean of normalized values of the MVA control group sera plus six standard deviations.

**Ethics statement**. The study was carried out in line with “The Code of Ethics of the World Medical Association” (Declaration of Helsinki). The use of serum samples complied with the guidelines of the Central Ethics Committee of the German Medical Association (Dtsch Arztebl 2003; 100(23): A-1632). In accordance with these guidelines, the anonymized use of residual material from samples sent to our laboratory for diagnostic purposes is permissible, provided that the patients have not decided against this procedure. Samples from patients who had decided against this procedure were excluded from the analysis. All animal experiments were handled in compliance with the European and national regulations for animal experimentation (European Directive 2010/63/EU; Animal Welfare Acts in Germany).

**Statistical analysis**. Data were prepared using GraphPad Prism 5 and are expressed as mean ± standard error of the mean ± interquartile range. Groups were compared using unpaired *t*-test or Mann–Whitney test when a large data spread was observed. A *p*-value of less than 0.05 was used as the threshold for statistical significance.

## Data Availability

The original contributions presented in the study are included in the article and Supplementary Materials, further inquiries can be directed to the corresponding author.

## Supplementary Materials

The following supporting information can be downloaded at: https://www.mdpi.com/article/10.3390/ijms252010898/s1. References [50,51,81,82] have been mentioned in the Supplementary Materials.
