# Supplementary material for: Implementation of an Immunoassay Based on the MVA-T7pol-Expression System for Rapid Identification of Immunogenic SARS-CoV-2 Antigens: A Proof-of-Concept Study
Source: Int J Mol Sci. 2024 Oct 10;25(20):10898. doi: 10.3390/ijms252010898 (PMC11508112; doi:10.3390/ijms252010898)
Supplement: Supplementary file 1 [file ijms-25-10898-s001.zip › ijms-3224099-supplementary.pdf]

International Journal of *Molecular Sciences*

MDPI

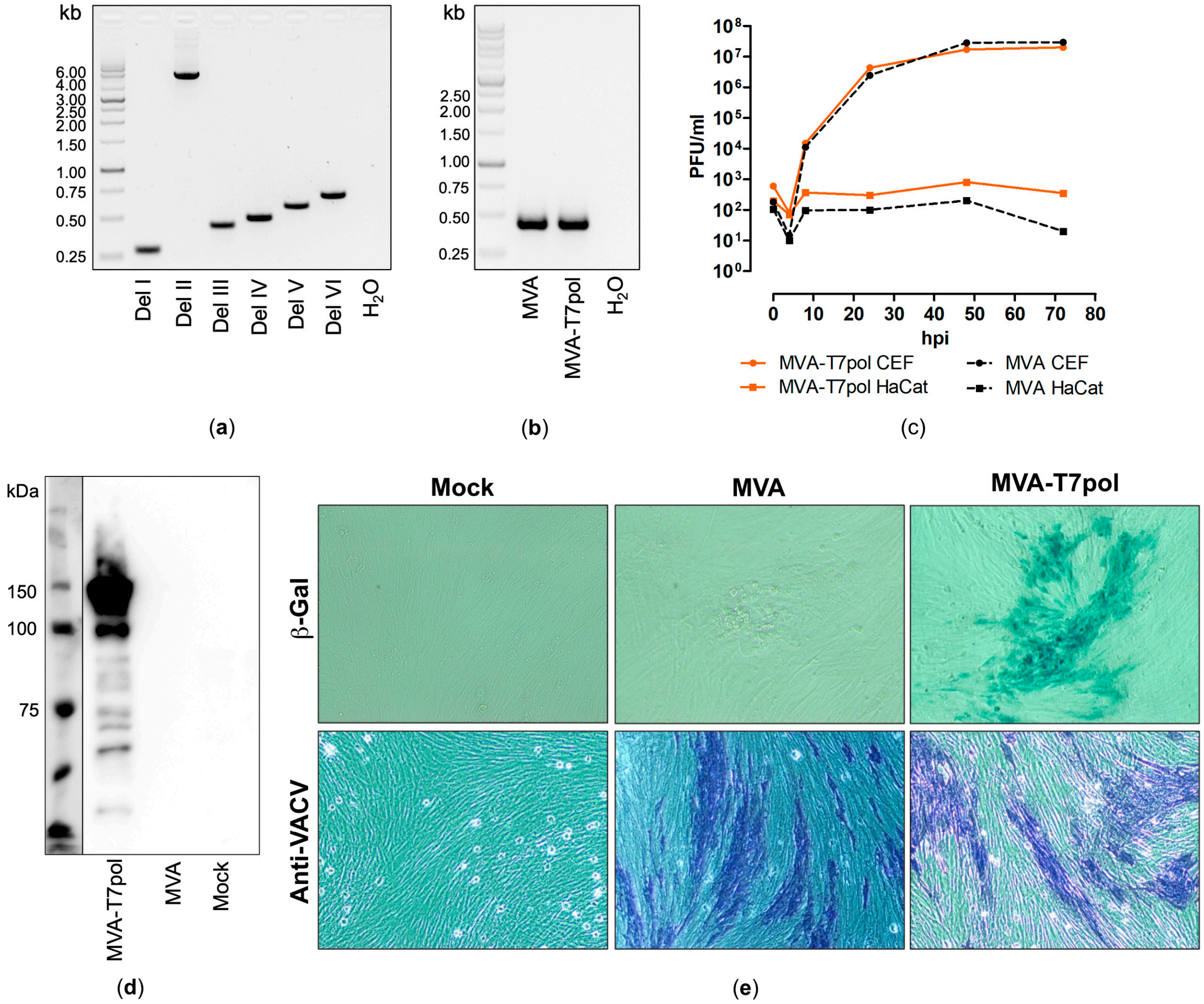

Figure S1. Virological characterization of MVA-T7pol. **(a, b)** Genetic stability of MVA-T7pol. **(a)** PCR analysis of genomic viral DNA demonstrated the genetic stability for six loci in the MVA-T7pol genome (deletion sites Del I-VI) including the heterologous T7pol/LacZ expression cassette inserted into the site of deletion II (Del II) with the amplification of a characteristic size DNA fragment (~5.0 kb). **(b)** PCR analysis of genomic viral DNA to monitor the *C7L* gene locus. PCR amplification of a specific 0.447 kb DNA fragment from the *MVA-C7L* gene sequence suggested the integrity of the *C7L* gene locus in the recombinant MVA-T7pol genome in comparison to the non-recombinant MVA (MVA) genome. The *C7L* gene is non-essential for MVA growth in chicken fibroblast cultures but the gene function is necessary to maintain unimpaired expression of MVA or recombinant genes under transcriptional control of vaccinia virus-specific late promoters [81]. **(c)** Multiple-step growth analysis of recombinant MVA-T7pol and non-recombinant MVA (MVA). Cells were infected at a multiplicity of infection (MOI) of 0.05 with MVA-T7pol or MVA and collected at the indicated time points. Titration was performed on CEF cells and plaque-forming units (PFU) were determined. MVA-T7pol and MVA could be efficiently amplified on CEF but failed to productively grow on human HaCat cells. **(d)** Western blot analysis of ß-Galactosidase in MVA-T7pol infected CEF cells. Cells were infected at a MOI of 5 and cell lysates were prepared 20 hpi. Non infected cells (Mock) and cells infected with non-recombinant MVA (MVA) served as controls. Polypeptides were resolved by SDS-PAGE and analyzed with an antibody against the ß-Galactosidase. **(e)** Detection of β-Galactosidase (β-Gal) activity in MVA-T7pol infected CEF cells. Cells were infected with MVA-T7pol and fixed 48 hpi. Non infected cells (Mock) and cells infected with non-recombinant MVA (Mock) served as controls. After fixation, cells were washed with PBS and stained with the staining solution following the manufacturer´s instructions. Subsequently, cells were washed with PBS and evaluated by microscopy. Additionally, cells were stained with an antibody directed against the vaccinia virus A27L protein (Anti-VACV) as control.

Table S1. Oligonucleotide sequences used for cloning the target regions

Primer	Sequence (5′➔3′)	Amplicon size (bp)	Target protein
N_{HA} forward	CAGCCCATGGGGTCTGATAATGGACCCCAAAATCAGCGAAATGCACCCCGCATTACG	1311	N
N_{HA} reverse	CTGCTGGTCGACTTATCAAGCGTAATCTGGAACATCGTATGGGTAGGCCTGAGTTGAGT		
E_{HA} forward	CAGCCCATGGGGTACTCATTCGTTTCGGAAGAGACAGGTACGTTAATAGTTAATAGCGTA	273	E
E_{HA} reverse	CTCGAGTTATCAAGCGTAATCTGGAACATCGTATGGGTAGACCAGAAGATCAGGAACTCT		
M_{HA} forward	CGACGAGGATCCATGGCAGATTCCAACGGTACTATTACCGTTGAAGAGCTTAAAAAGCTC	717	M
M_{HA} reverse	CTCGAGTTATCAAGCGTAATCTGGAACATCGTATGGGTACTGTACAAGCAAAGCAATATT		
$ORF3a_{HA}$ forward	CAGCCCATGGATTTGTTTATGAGAATCTTCACAATTGGAACTGTAACTTTGAAGCAAGGA	870	ORF3a
$ORF3a_{HA}$ reverse	CTCGAGTTATCAAGCGTAATCTGGAACATCGTATGGGTACAAAGGCACGCTAGTAGTCGT		
$ORF6_{HA}$ forward	CAGCCCATGGGGTTTCATCTCGTTGACTTTCAGGTTACTATAGCAGAGATATTACTAATT	231	ORF6
$ORF6_{HA}$ reverse	CTCGAGTTATCAAGCGTAATCTGGAACATCGTATGGGTAATCAATCTCCATTGGTTGCTC		
$ORF7a_{HA}$ forward	CAGCCCATGGGGAAAATTATTCTTTTCTTGGCACTGATAACACTCGCTACTTGTGAGCTT	411	ORF7a
$ORF7a_{HA}$ reverse	CTCGAGTTATCAAGCGTAATCTGGAACATCGTATGGGTATTCTGTCTTTCTTTTGAGTGTG		
$ORF8_{HA}$ forward	CAGCCCATGGGGAAATTTCTTGTTTTCTTAGGAATCATCACAACTGTAGCTGCATTTCAC	411	ORF8a
$ORF8_{HA}$ reverse	CTCGAGTTATCAAGCGTAATCTGGAACATCGTATGGGTAGATGAAATCTAAAACAACACG		

The restriction enzyme sites are boxed (NcoI: CCATGG; SalI: GTCGAC; XhoI: CTCGAG; BamHI: GGATCC). The HA sequences are underlined.

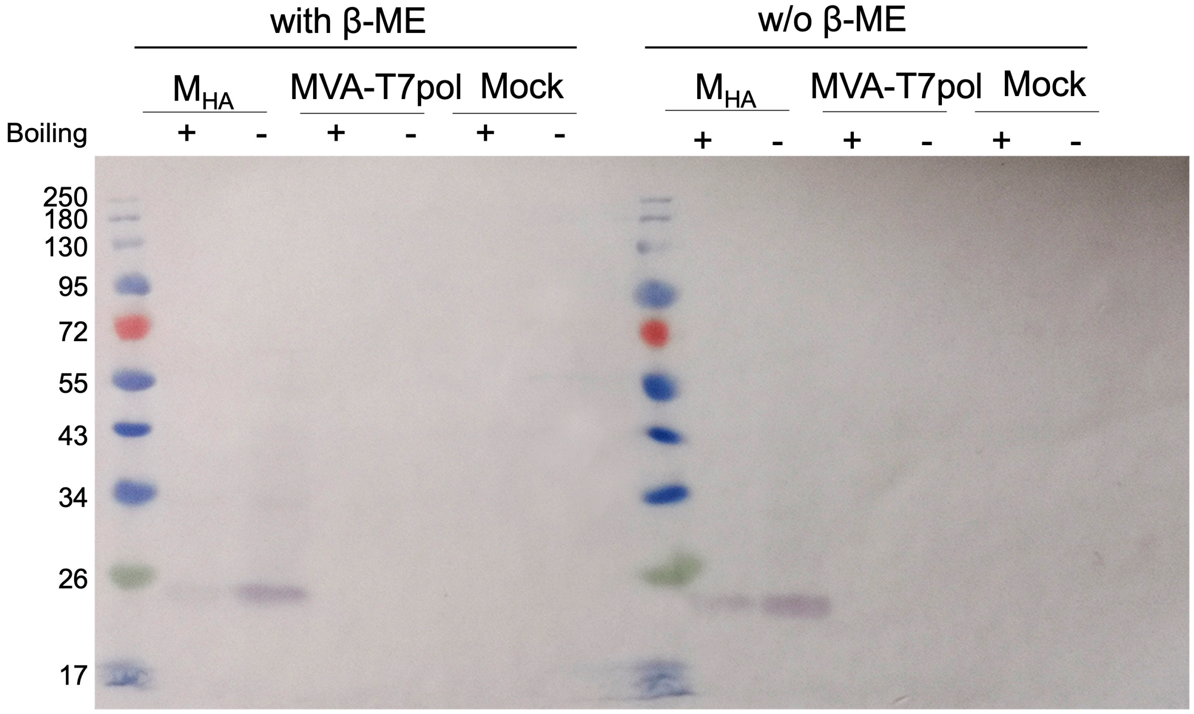

Figure S2. Comparison of different sample treatment conditions to enhance the signal of recombinant SARS-CoV-2 membrane protein (M_{HA}). CEF cells were infected with MVA-T7pol at a MOI of 10 and transfected with pOS6-M_{HA}. Non-infected cells (Mock) and cells infected with MVA-T7pol served as controls. 24 h post infection, cells were harvested, and lysates were prepared by adding lysis buffer mixed with Laemmli sample buffer supplemented with or without (w/o) β-mercaptoethanol (β-ME). The samples were treated at 95°C (+) or room temperature (-) for 5 min. After electrophoresis and transfer, the specific proteins were identified by using an antibody directed against the HA-tag. The observed bands for M_{HA} (~26 kDa) without boiling were sharper than boiled ones, regardless of whether adding β-ME or not.

Table S2. SARS-CoV-2 specific antibody responses in COVID-19 patient analyzed by Western Blot, ELISA and SNT

Patient No.	Western blot[a]	IgG-ELISA	IgA-ELISA	SNT
1	N (++), S (++), ORF3a (+)	positive	positive	>80
2	N (++), S (++)	positive	positive	>80
3	N (++), S (+)	positive	positive	>80
4	N (+), S (+)	positive	positive	40
5	N (+), S (+)	positive	positive	40
6	N (+), S (+)	positive	positive	40
7	N (+), S (-)	negative	negative	negative
8	N (+), S (-)	negative	positive	negative
9	N (-), S (-)	negative	negative	negative

[a] -, negative signal; +, weak positive signal; ++, strong positive signal

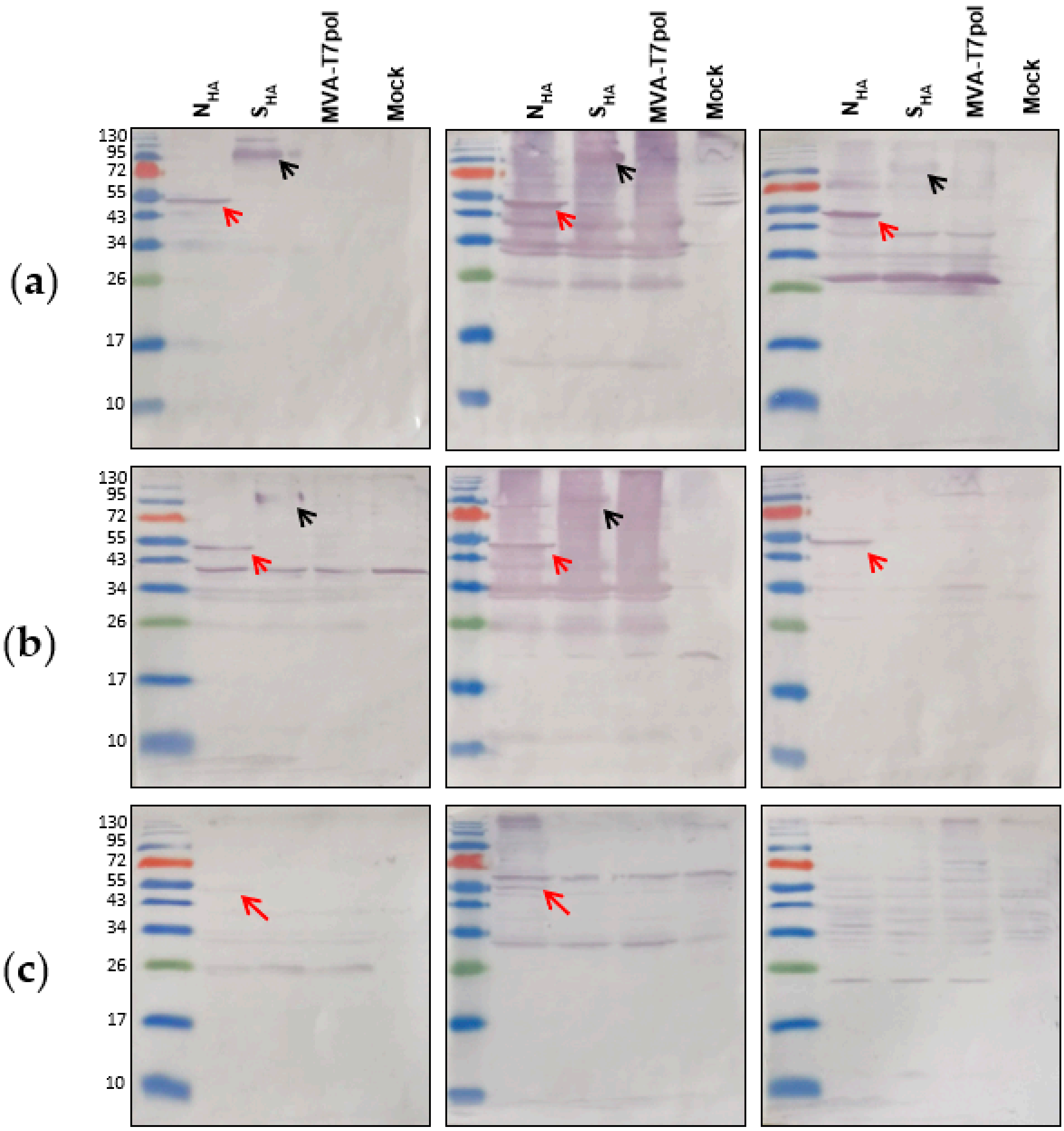

Figure S3. Detection of SARS-CoV-2 antibodies in patient sera using the MVA-T7pol expression system. CEF cells were either infected with MVA-T7pol at a MOI of 10 and transfected with vector plasmid pOS6-N_{HA} or infected with MVA-S_{HA} at a MOI of 10. Non-infected cells (Mock) and cells infected with MVA-T7pol served as controls. Cells were lysed and proteins were separated by SDS-PAGE followed by Western Blot analysis with patient sera. The bands representing the presence of antibodies against N are marked by red arrows; the bands representing the presence of antibodies against S are marked by black arrows. (**a**) three different patient sera (#1-3) from group 1 (SNT: >1:80), (**b**) three different patient sera (#4-6) from group 2 (SNT: 1:40), **(c)** three different patient sera (#7-9) from group 3 (no detectable neutralizing antibodies).

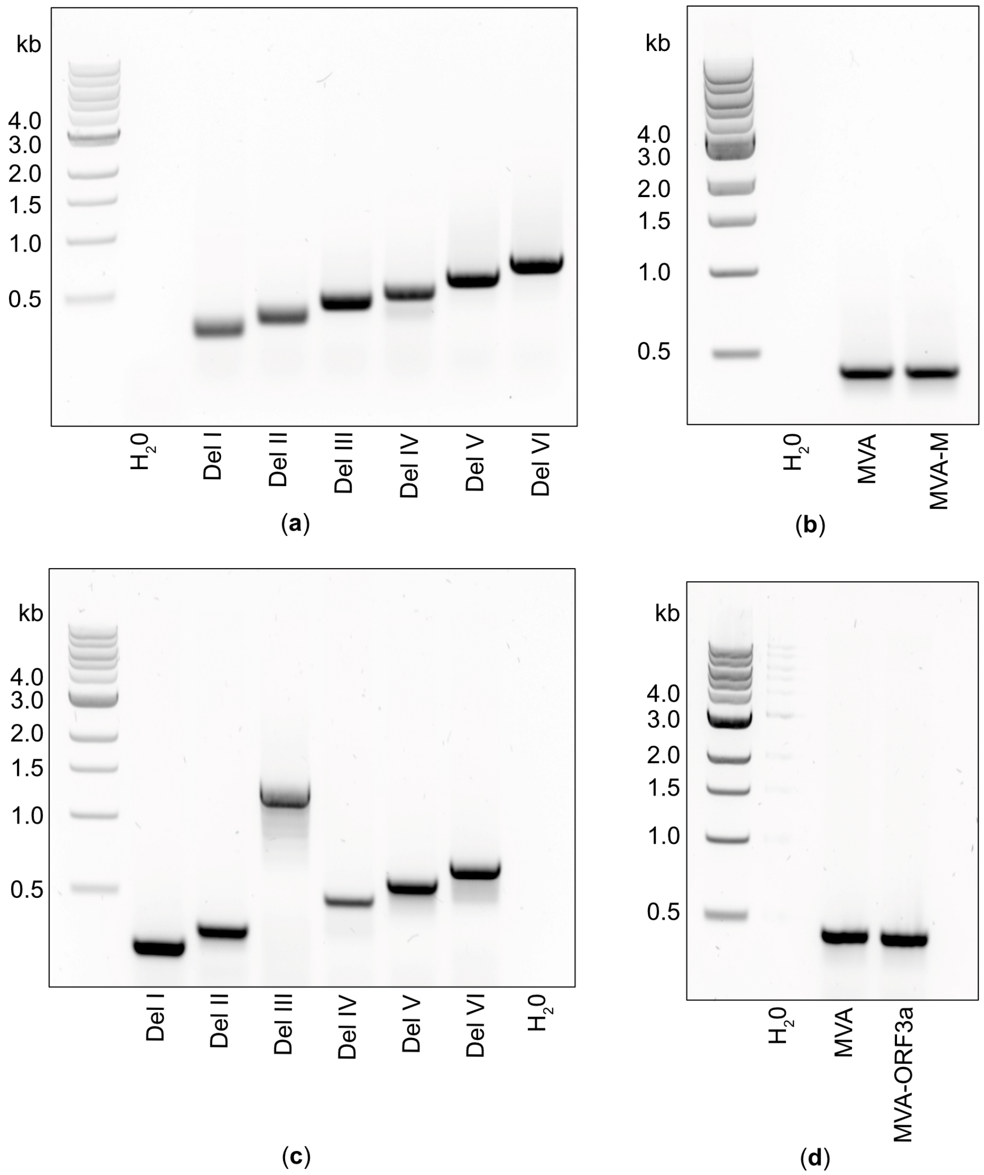

Figure S4. Virological characterization of MVA-M and MVA-ORF3a. (a, b) Genetic integrity of MVA-M. PCR analysis of viral DNA to monitor (**a**) the genetic stability for six loci in the MVA-M genome (deletion sites Del I-VI) and **(b)** the *C7L* gene locus. **(c, d)** Genetic integrity of MVA-ORF3a. PCR analysis of viral DNA to monitor **(c)** the genetic stability for six loci in the MVA-ORF3a genome (deletion sites Del I-VI) including the heterologous SARS-CoV-2-ORF3a gene sequence inserted into the site of deletion III (Del III) and (**d**) the *C7L* gene locus.

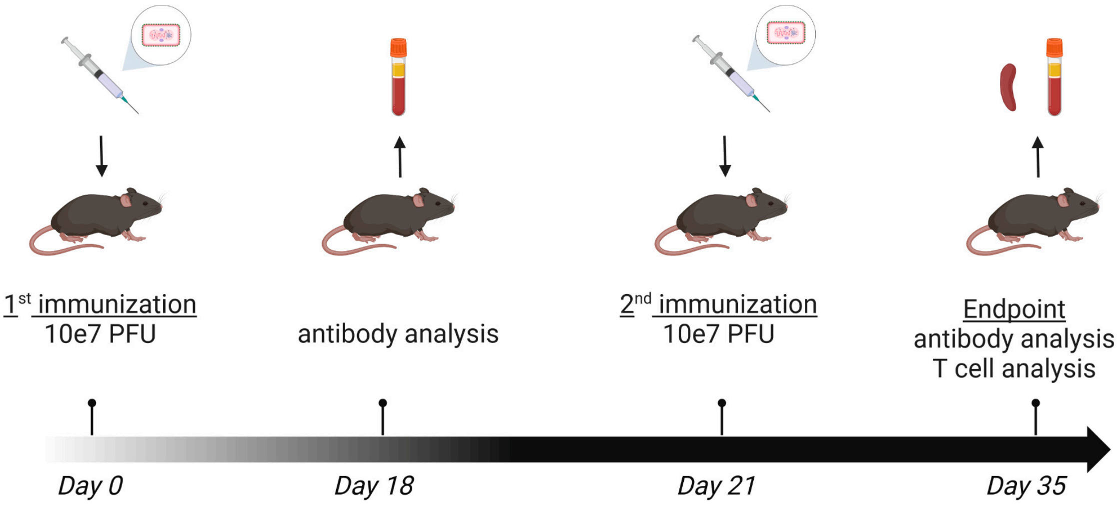

(a)

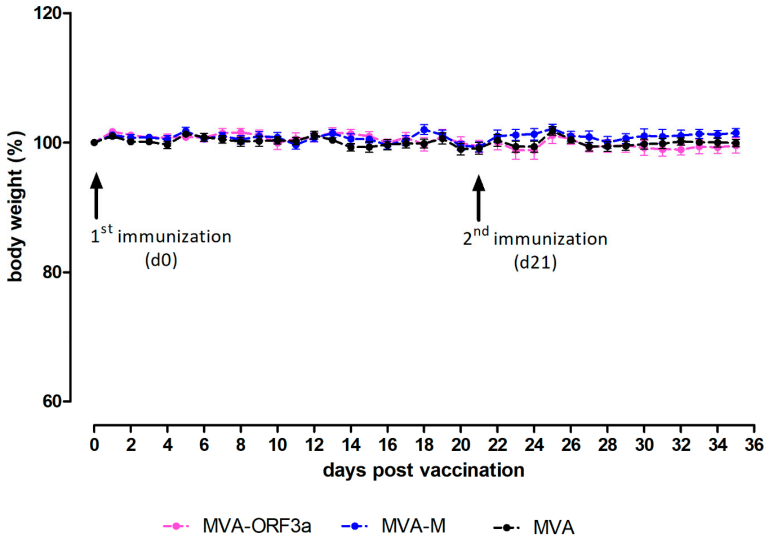

(b)

Figure S5. MVA-ORF3a and MVA-M immunization schedules and monitoring for side effects. Groups of *HLA-A2.1-/HLA-DR1-transgenic H-2 class I-/class II-knockout* mice were vaccinated with 10^7 PFU MVA-ORF3a and MVA-M via the intramuscular route using a prime-boost schedule (21-day interval). **(a)** Schematic diagram of the immunization schedules. Antibody responses were tested at days 18 and 35 post prime immunization. T cell responses were tested at day 35 post prime immunization. **(b)** Body weight changes after prime-boost vaccination with MVA-ORF3a and MVA-M. Body weight and clinical score was measured daily. No severe side effects were observed.

Table S3. Predicted SARS-CoV-2-ORF3a peptides used for ELISPOT and ICS-FACS

Name	Sequence	Reference
ORF3a$_{72-80}$	ALSKGVHFV	[50,82]
ORF3a$_{82-90}$	NLLLLFVTV	[50,51]
ORF3a$_{107-115}$	YLYALVYFL	[50]
ORF3a$_{137-147}$	NPLLYDANYFL	[50]

Table S4: SARS-CoV-2-M overlapping peptides used for ELISPOT

Name	Sequence	Pool
M1	MADSNGTITVEELKK	Pool 1
M2	NGTITVEELKKLLEQ	Pool 1
M3	TVEELKKLLEQWNLV	Pool 1
M4	LKKLLEQWNLVIGFL	Pool 1
M5	LEQWNLVIGFLFLTW	Pool 1
M6	NLVIGFLFLTWICLL	Pool 1
M7	GFLFLTWICLLQFAY	Pool 2
M8	LTWICLLQFAYANRN	Pool 2
M9	CLLQFAYANRNRFLY	Pool 2
M10	FAYANRNRFLYIIKL	Pool 2
M11	NRNRFLYIIKLIFLW	Pool 2
M12	FLYIIKLIFLWLLWP	Pool 2
M13	IKLIFLWLLWPVTLA	Pool 3
M14	FLWLLWPVTLACFVL	Pool 3
M15	LWPVTLACFVLAAVY	Pool 3
M16	TLACFVLAAVYRINW	Pool 3
M17	FVLAAVYRINWITGG	Pool 3
M18	AVYRINWITGGIAIA	Pool 3
M19	INWITGGIAIAMACL	Pool 4
M20	TGGIAIAMACLVGLM	Pool 4
M21	AIAMACLVGLMWLSY	Pool 4
M22	ACLVGLMWLSYFIAS	Pool 4
M23	GLMWLSYFIASFRLF	Pool 4
M24	LSYFIASFRLFARTR	Pool 4
M25	IASFRLFARTRSMWS	Pool 5
M26	RLFARTRSMWSFNPE	Pool 5
M27	RTRSMWSFNPETNIL	Pool 5
M28	MWSFNPETNILLNVP	Pool 5
M29	NPETNILLNVPLHGT	Pool 5
M30	NILLNVPLHGTILTR	Pool 5
M31	NVPLHGTILTRPLLE	Pool 6
M32	HGTILTRPLLESELV	Pool 6
M33	LTRPLLESELVIGAV	Pool 6
M34	LLESELVIGAVILRG	Pool 6
M35	ELVIGAVILRGHLRI	Pool 6
M36	GAVILRGHLRIAGHH	Pool 6
M37	LRGHLRIAGHHLGRC	Pool 7
M38	LRIAGHHLGRCDIKD	Pool 7
M39	GHHLGRCDIKDLPKE	Pool 7
M40	GRCDIKDLPKEITVA	Pool 7
M41	IKDLPKEITVATSRT	Pool 7
M42	PKEITVATSRTLSYY	Pool 7
M43	TVATSRTLSYYKLGA	Pool 8

M44	SRTLSYYKLGASQRV	Pool 8
M45	SYYKLGASQRVAGDS	Pool 8
M46	LGASQRVAGDSGFAA	Pool 8
M47	QRVAGDSGFAAYSRY	Pool 8
M48	GDSGFAAYSRYRIGN	Pool 8
M49	FAAYSRYRIGNYKLN	Pool 9
M50	SRYRIGNYKLNTDHS	Pool 9
M51	IGNYKLNTDHSSSSD	Pool 9
M52	KLNTDHSSSSDNIAL	Pool 9
M53	DHSSSSDNIALLVQ	Pool 9

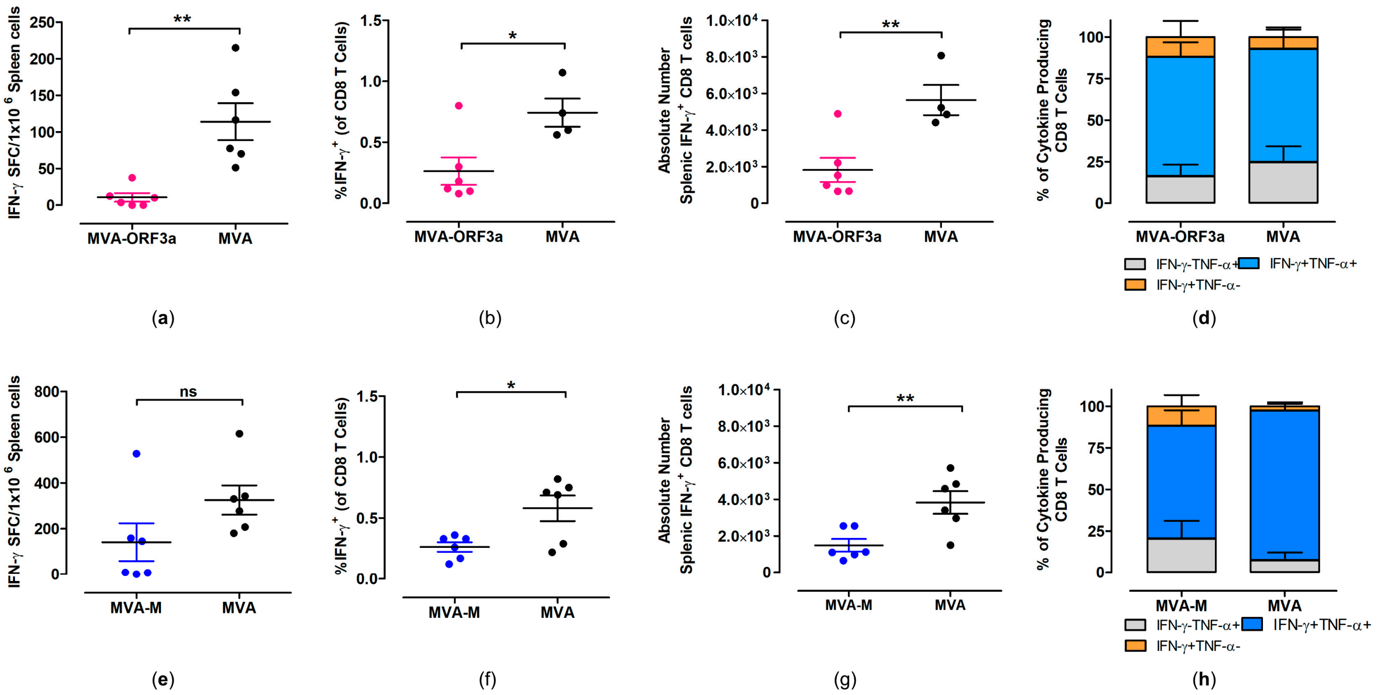

Figure S6. Activation of MVA-specific CD8+ T cells after prime-boost vaccination with MVA-M and MVA-ORF3a. Groups of *HLA-A2.1-/HLA-DR1-transgenic H-2 class I-/class II-knockout* mice were immunized with 10^7 PFU of MVA-M and MVA-ORF3a via the i.m. route. Mice immunized with non-recombinant MVA (MVA) served as controls. Splenocytes were collected and prepared on day 14 after booster immunization. Splenocytes were stimulated with the H2d restricted MVA-specific peptide $A6(L)_{6\text{-}14}$ and measured by IFN-γ ELISPOT assay (**a, e**) and intracellular cytokines staining (ICS) plus FACS analysis (**b-d, f-g**). (**a, e**) IFN-γ spot-forming colonies (SFC) measured by ELISPOT assay. (**b, c, f, g**) IFN-γ producing CD8+ T cells measured by FACS analysis. Graphs show the mean frequency and absolute number of IFN-γ+ CD8 T cells. (**d, h**) Cytokine profile of $A6_{6\text{-}14}$ specific CD8 T cells. Graph show the mean frequency of IFN-γ^-TNF-α^+, IFN-γ^+TNF-α^+, and IFN-γ^+TNF-α^- cells within the cytokine positive CD8 T cell compartment. Bars represent the mean + standard error of the mean (SEM). Difference between group were analyzed by unpaired, two-tailed t test * $p < 0.05$, ** $p < 0.01$.

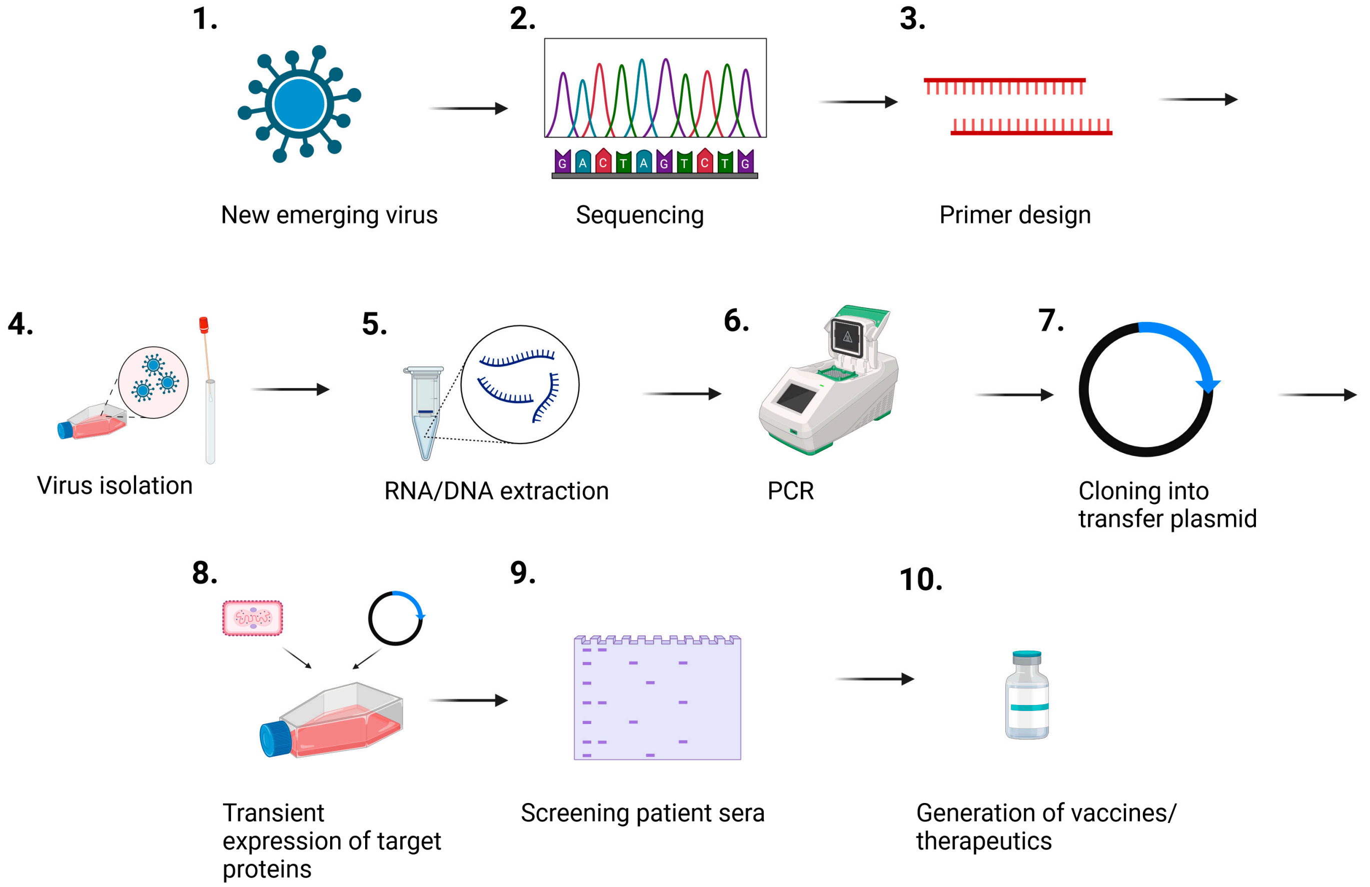

Figure S7: Workflow immunoassay based on the MVA-T7pol expression system. New emerging viruses are sequenced to identify coding regions of viral proteins. Based on the sequencing results, oligonucleotide sequences targeting full-length viral proteins/target regions are designed, including restriction enzyme sites for cloning into the transfer plasmid. The virus is isolated (e.g. from cell cultures, swabs, stool samples, urine samples) and RNA/DNA is extracted. If using RNA, an additional reverse transcription step is need to generate cDNA. Next, PCR is performed to amplify the target regions, using the designed oligonucleotide sequences. Afterwards, sequences are cloned into the transfer plasmid (e.g. pTM3, pOS6) and cell cultures are infected with MVA-T7pol and transfected with the transfer plasmid to overexpress the proteins. Subsequently, Western Blot analysis is performed and patient sera are screened for the presence of antibodies directed against the viral proteins. Based on the results, vaccines or therapeutics can be generated and further evaluated in preclinical studies. Created with BioRender.com.

Table S5: Antibodies and staining reagent for flow cytometry

Antigen	Fluorophore	Company	Catalogue No.
CD3	PE-Cy7	BioLegend,	503832
CD4	Brilliant Violet 421	BioLegend	100437
CD8α	Alexa Fluor 488	BioLegend	100723
CD16/CD32	n/a	BioLegend	101320
IFN-γ	APC	BioLegend	505810
TNF-α	PE	BioLegend	506306

References

50. Saini, S.K.; Hersby, D.S.; Tamhane, T.; Povlsen, H.R.; Amaya Hernandez, S.P.; Nielsen, M.; Gang, A.O.; Hadrup, S.R. SARS-CoV-2 genome-wide T cell epitope mapping reveals immunodominance and substantial CD8+ T cell activation in COVID-19 patients. *Sci. Immunol.* **2021**, *6*, doi:10.1126/sciimmunol.abf7550.
51. Jin, X.; Liu, X.; Shen, C. A systemic review of T-cell epitopes defined from the proteome of SARS-CoV-2. Virus Res. 2022, 324, 199024. https://doi.org/10.1016/j.virusres.2022.199024.
81. Backes, S.; Sperling, K.M.; Zwilling, J.; Gasteiger, G.; Ludwig, H.; Kremmer, E.; Schwantes, A.; Staib, C.; Sutter, G. Viral host-range factor C7 or K1 is essential for modified vaccinia virus Ankara late gene expression in human and murine cells, irrespective of their capacity to inhibit protein kinase R-mediated phosphorylation of eukaryotic translation initiation factor 2alpha. *J. Gen. Virol.* **2010**, *91*, 470–482, doi:10.1099/vir.0.015347-0.
82. Kared, H.; Redd, A.D.; Bloch, E.M.; Bonny, T.S.; Sumatoh, H.; Kairi, F.; Carbajo, D.; Abel, B.; Newell, E.W.; Bettinotti, M.P.; et al. SARS-CoV-2-specific CD8+ T cell responses in convalescent COVID-19 individuals. *J. Clin. Invest.* **2021**, *131*, doi:10.1172/JCI145476.
